# Effects of Chronic Interpersonal Stress Exposure on Depressive Symptoms are Moderated by Genetic Variation at *IL6* and *IL1β* in Youth

**DOI:** 10.1016/j.bbi.2015.01.003

**Published:** 2015-01-13

**Authors:** Margaret Tartter, Constance Hammen, Julienne E. Bower, Patricia A. Brennan, Steven Cole

**Affiliations:** a Department of Psychology, University of California, Los Angeles; b Department of Psychiatry and Biobehavioral Sciences, University of California, Los Angeles; c Cousins Center for Psychoneuroimmunology, University of California, Los Angeles; d Department of Psychology, Emory University, Atlanta, GA, USA; e School of Medicine, University of California, Los Angeles

**Keywords:** Pro-inflammatory cytokines, depression, adolescent depression, *IL1β*, *IL6*, *TNF*, gene-environment interaction

## Abstract

**Aims:**

Close to one third of patients with major depression show increases in pro-inflammatory cytokines, which are in turn associated with risk for inflammatory disease. Genetic variants that enhance immune reactivity may thus enhance inflammatory and depressive reactions to stress. The aim of the present study was to investigate a trio of functional SNPs in the promoter regions of *IL6* (-174G>C, rs1800795), *IL1β* (-511C>T, rs16944), and *TNF* (-308G>A, rs1800629) as moderators of the relationship between chronic stress exposure and elevations in depressive symptoms.

**Methods:**

Participants were 444 Australian youth (mean age = 20.12) whose exposure to chronic stress in the past 6 months was assessed using the semi-structured UCLA Life Stress Interview, and who completed the Beck Depression Inventory II at ages 15 and 20. Between ages 22 and 25, all participants in the selected sample provided blood samples for genotyping.

**Results:**

In line with a hypothesized moderation effect, -174G allele carriers at *IL6* had *fewer* depressive symptoms following interpersonal stress, relative to C/C homozygotes with equal interpersonal stress exposure. However, *IL6* genotype did not moderate the effects of non-interpersonal stress exposure (i.e., financial, work and health-related difficulties) on depression. Also in line with hypotheses, the -511C allele in *IL1β*, previously associated with higher IL-1β expression, was associated with more severe depression following chronic interpersonal stress exposure, relative to T/T homozygotes. Again, the moderating effect was specific to interpersonal stressors and did not generalize to non-interpersonal stress. *TNF* was not a moderator of the effects of either interpersonal or non-interpersonal stress on later depression outcomes.

**Conclusion:**

Findings were consistent with the hypothesis that pro-inflammatory genetic variation increases the risk of stress-induced depression. The present results provide evidence of a genetic mechanism contributing to individual differences in depressive symptomatology following interpersonal stress exposure.

## 1. Introduction

Depression is moderately heritable and often manifests following exposure to psychosocial stress (Kendler et al., 1995; Kendler, Thornton & Gardner, 2001). Activation of the inflammatory response, measured by increased plasma levels of pro-inflammatory cytokines, including interleukin-1β (IL-1β), interleukin-6 (IL-6), tumor-necrosis factor alpha (TNF-α), and C-reactive protein (CRP), has been shown to follow exposure to psychosocial stress, and is associated with rises in depressive symptoms and with major depressive disorder (Dowlati et al., 2010; Howren et al., 2009; Matthews et al., 2007; Matthews et al., 2010; Miller and Cole, 2012; Pasco et al., 2010; Raison and Miller, 2011; Van Den Biggelaar et al., 2007). Genetic variants that enhance immune reactivity may enhance depressive reactions to stress, creating vulnerability to depressive disorders. To our knowledge, the present study is the first to examine the interaction between stress exposure and polymorphisms of the inflammatory response as a predictor of depressive symptoms.

Interpersonal stress strongly elicits depressive symptoms, and also increases proinflammatory cytokine production (Dickerson et al., 2009; Monroe et al. 1999; Slavich et al. 2010a, 2010b). Chronic exposure to interpersonal stress is associated with persistent and unhealthy inflammatory activity (Dhabhar, 2014, 2009; Miller et al., 2009). For instance, older adults who endorse high levels of loneliness show increased expression of pro-inflammatory genes and decreased expression of anti-inflammatory factors in leukocytes, compared to those who feel socially integrated (Cole et al., 2007.

A functional SNP in *IL6* (-174G>C) has received attention in the literature for its effects on interpersonal stress-related *IL6* expression (Cole et al., 2010; Schultz-Florey et al., 2012). Possession of a G allele at -174 increases expression of IL-6 mRNA and plasma levels of IL-6, following exposure to the stress hormone norepinephrine, and also in response to grief in midlife and older adults (Cole et al., 2010; Schultz-Florey et al., 2012).

Of developmental relevance to our study, this locus may have opposite effects on cytokine expression in response to interpersonal stressors in youth. In a study of 17-19 year olds exposed to varying levels of psychosocial stress, those with CC genotypes had greater plasma CRP, a marker of chronic IL-6 production, compared to GG or GC genotypes (Cole et al., 2011). One interpretation of the changing allele of risk by age is that the G allele provides protection against inflammation prior to reproductive age, but becomes sensitized to stress during the transition to adulthood, likely reflecting evolutionary adaptations to promote survival early on in life (Cole et al., 2010; Williams, 1957).

Although not previously investigated as moderators, *TNF* -308G>A has been directly linked to depressive symptoms in women with breast cancer, a context likely to include stress exposure, and to a higher rate of attempted suicide among patients with MDD (Bower et al., 2013; J. Kim et al., 2013; Y. Kim et al., 2013, but see Lotrich, Ferrell, Rabinovitz & Pollock, 2010, for non-significant relationship to depression in interferon-alpha patients), and *IL1β* -511C>T has been found to predict failure to remit after antidepressant treatment (Baune et al., 2010). Stress exposure was not measured in these investigations, and to our knowledge no study has undertaken a gene-environment approach to understanding the impact of these loci on risk for depression.

The primary aim of the present study was to examine *IL6* (-174G>C, rs1800795; Smith and Humphries, 2009) as a moderator of depressive reactions to chronic stress exposure in a large cohort of adolescents, given the relevance of *IL6* to stress-induced inflammation and mood. We were also interested in examining *IL1β* (−511C>T, rs 16944, Dixon et al., 2007) and *TNF* (-308G>A, rs1800629, Abraham and Kroeger, 1999; McGuire et al., 1994; Nadel et al., 1996, but see Bayley et al., 2004) as moderators of the stress-depression relationship, based on their association with changes in pro-inflammatory cytokine expression, and preliminary evidence of their relevance to mood symptoms. Given the young age of the present sample, and the findings of Cole et al. (2010), we hypothesized that the C allele in *IL6* would confer risk for stress-induced changes in depression. We hypothesized that the high expression variants of *IL1β* and *TNF* would potentiate increases in depressive symptoms following exposure to chronic stress. We were particularly interested in whether genetic moderation would be specific to chronic interpersonal stress, or would generalize to non-interpersonal stressors as well.

## 2. Method

### 2.1 Participants

Participants were 444 young adults, ages 22-25, drawn from the much larger University of Queensland Study of Pregnancy (MUSP) birth cohort, based on their participation in a genetic substudy. The parent study enrolled 7,223 publicly supported maternity patients and their children born at Mater Misericordiae Mothers' Hospital, in Brisbane, Australia between 1981 and 1984 (Keeping et al., 1989; see Table 1). Mothers in the original MUSP cohort completed self-report questionnaires that measured symptoms of depression using the Delusions-Symptoms States Inventory (DSSI; Bedford and Foulds, 1978) during pregnancy, at birth, and at child ages 6 months and 5 years (see Najman et al., 2005, for full details of the sample selection). When the children turned 15 years old, a sample of 815 families was selected, oversampling for maternal depression, for a substudy of outcomes in offspring of depressed and never-depressed mothers. Further details concerning the selection of this sample are provided in Hammen and Brennan, 2001.

Participants in the selected sample were re-contacted when youth turned 20 for additional psychological assessment, and 706 mother-child pairs completed the age 20 data collection. Between the ages of 22 and 25, the 815 youth who participated in the age 15 assessment were invited to provide blood samples for genetic analyses. Failure to respond to requests for participation, medical problems, death and invalid DNA samples resulted in a total of 444 participants who contributed blood samples for the genetic data collection, and the current study sample is limited to these individuals, focusing on psychosocial measures taken at ages 15 and 20, supplemented by health survey data collected in early life that assessed the presence or absence of asthma. This final sample was evenly split by gender (49.4% female), predominantly Caucasian (92.1%), and came from lower and lower-middle income homes. Non-Caucasian participants were Asian (4.1%), Maori/Pacific Islander (1.1%), Australian Aborigine (2%) and other (0.2%). Those who completed the genetic study were more likely to be female (χ^2^(1) = 33.66, *p* < .001), but were no more likely to have mothers with a history of depression (χ^2^(1) = .013, *n.s*.) compared to youth that did not complete the genetic study. There were no differences in ethnicity (χ^2^(5) = 6.40, *n.s*.), depressive symptoms at ages 15 or 20 (*t*(631) = -1.44, *n.s*; *t*(803) = -.83, *n.s*.), or in chronic stress exposure in the 6 months prior to age 20 (interpersonal: *t*(813) = -1.97, *n.s*; non-interpersonal: *t*(813) = 1.26, *n.s*), between the overall sample and the genetic subsample.

### 2.2 Procedure

Mothers and children in the sample completed the Structured Clinical Interview for DSM-IV (SCID) and self-report measures of symptoms, social functioning, and family environment when offspring turned 15, and again when they turned 20. Information was gathered in the family home or a location convenient for the participants and interviewer. Postgraduate psychology students were trained to appropriately conduct and reliably score these interviews. Participants all gave informed consent, or assent in the case of minors. The institutional review or ethics panels of the University of Queensland, University of California, Los Angeles, and Emory University approved the research protocol.

Youth were contacted in 2006, between ages 22 and 25, regarding participation in a blood draw for genotyping purposes. Those who wished to participate in the genotyping study were mailed consent forms, a blood collection kit, and questionnaires. They were instructed to have a blood draw completed by a local phlebotomist, and the samples were then picked up by courier and transported to the Genetic Epidemiological Laboratory of the Queensland Institute of Medical Research (QIMR). DNA extraction from leukocytes took place via the salting out method (Miller et al., 1988). The resultant “stock” DNA was eluted in 400µl of 1 × Tris-EDTA buffer (10mM Tris pH 8.0, 1mM EDTA pH 8.0) and ranged in concentration from 100ng/μl -100μg/μl. DNA samples were stored at the QIMR laboratory until 2011, when aliquots of DNA were sent to the UCLA Social Genomics Core Laboratory for genotyping. Allelic variation at *IL6* (G > C; rs1800795), *IL1β* (-511 C > T; rs16944) and *TNF* (-308 G >A; rs1800629) was assayed by a commercial TaqMan Genotyping Assay (Applied Biosystems, Foster City, CA) performed on an iCycler real-time PCR instrument (BioRad, Hercules, CA) following the manufacturer's specified protocol (Cole et al., 2010).

Test-retest reliability of duplicated specimens yielded a total genotyping error rate below 1%. All three polymorphisms were in Hardy-Weinberg equilibrium (χ^2^ (1, 444) values: *IL1β* -511 = 0.26, *IL6* -174 = 0.13, *TNF* -308 = 0.08, all *p*-values > .05). Population level minor allele frequencies of the three SNPs were 18.5% at *IL6*, 46.5% at *IL1β*, and 9.6% at *TNF*, indicating that allelic variation at these loci is common, and could reasonably account for differences in depressive symptoms.

### 2.3 Measures

#### 2.3.1 Depression symptoms

Youth self-report of depression symptoms was collected at ages 15 and 20 using the Beck Depression Inventory-II (BDI-II; Beck et al., 1996), a continuous measure that has shown excellent internal consistency, and has been validated against clinical ratings of depression (Beck et al., 1988). Internal consistency was very good in the present sample (Crohnbach's α =.90). Scores on the BDI-II at age 15 ranged from 0 to 32 (M = 6.20, SD = 6.19). At age 20, scores ranged from 0-52 (M = 7.44, SD = 8.40; see Table 1). Use of a continuous measure of depressive symptoms provides a sensitive test of the present study's gene-environment interaction hypotheses, while the use of diagnostic categories of depressive disorders would greatly reduce the ability to detect effects. Further, diagnostic categories of depressive disorders have shown only slight advantage over subsyndromal depressive symptoms as indicators of functional impairment (Judd et al., 1996).

#### 2.3.2 Chronic Interpersonal Stress

Youth were interviewed at age 20 using the semi-structured UCLA Life Stress Interview (LSI; Hammen and Brennan, 2001), which was developed to assess chronic, ongoing stressful conditions in major life domains, as well as acutely stressful life events, and has been validated for use in young adult samples (Adrian and Hammen, 1993; Rao et al., 1999). Using a set of standardized questions and follow up prompts, interviewers asked participants to describe their functioning in eight different life domains, half of them related to social functioning, over the past six months. Functioning in each domain was then scored on a scale of 1 to 5, with half-points permitted, where 1 indicates superior functioning and 5 indicates significantly impaired functioning. For example, in the Romantic Relationship domain, a score of 1 would be given to an individual who has a stable romantic relationship that is low in conflict and high in self-disclosure, and who engages in frequent positive interactions with their partner. An individual who reports a close relationship that is lacking in disclosure, or who experiences regular conflict in their relationship would score a 3. An individual who reports isolation from their partner or severe or frequent conflict with their partner would receive a score of 5. The four domains with social content (romantic relationships, relationship with a best friend, family relationships, and social life) were then summed to create a composite score reflecting chronic interpersonal stress, where higher scores indicate greater distress. The remaining four domains (work, finances, personal health, family health) were summed to create a composite measure of non-interpersonal chronic stress. Composite scores across interpersonal domains ranged from 4 to 18.5 with a mean of 10.04 (SD = 2.59). Composite scores across non-interpersonal domains ranged from 3 to 15 with a mean of 7.59 and a standard deviation of 2.02.

#### 2.3.3 Maternal Depression

Mothers were interviewed when their child turned 15, using the Structured Clinical Interview for DSM-IV (SCID-IV; First et al., 1995). Nearly half (43.5%) of the mothers in this high-risk sample met criteria for a current or past episode of major depression or dysthymic disorder by youth age 15.

### 2.4 Statistical Analysis

All statistical tests were two-sided and conducted using SPSS statistical package, version 18.0 for Macintosh. The simple effects of significant interactions were examined in Stata 12.0 for Macintosh. All stress variables were mean-centered to reduce multicollinearity, and missing data (3 cases at age 15, and 6 cases at 20) were replaced with the variable mean. Maternal depression was entered as a covariate in all analyses, as depression in the mother has been linked to increased exposure to psychosocial stress and also contributes heritable risk for depression. Additionally, gender and scores on the BDI-II at age 15 were entered as covariates to insure that earlier depressive symptoms, especially among women, did not better account for current symptoms. Childhood asthma was the most common inflammatory illness based on health diagnoses recorded at age 5, and was entered as a covariate to control for possible confounding effects of heightened inflammation on depression.

Linear regression was used to test stress exposure in the past 6 months, inflammatory genotypes, and the interactions of stress and genotype, as predictors of depressive symptoms at age 20. All genotypes were coded as three-level variables (0 = minor allele homozygotes, 1 = heterozygotes, 2 = major allele homozygotes). Post-hoc contrasts were planned to evaluate the simple effects of significant interactions between genotype and stress. At *IL1β* and *TNF*, contrasts evaluated the hypothesis that dominant homozygotes would have greater levels of depression compared to heterozygotes and to recessive homozygotes. In adolescents, the allele of risk at *IL6* has been identified as -174C, and post-hoc contrasts compared CC homozygotes to CG and GG genotypes. In light of the conflicting findings regarding the allele of risk at *IL6*, we also compared CG and GG genotypes. Given the a priori hypotheses and exploratory nature of the analyses, correction for multiple testing was not planned. Following the primary analyses, the models were re-run restricting the sample to Caucasians, the largest ethnic group, to examine the impact of ethnicity on genetic effects.

## 3. Results

We first tested the specificity of chronic interpersonal stress as a predictor of depressive symptoms. Both interpersonal and non-interpersonal stress remained significant when entered in a single model predicting age 20 BDI-II score (*b* = 0.92, SE = 0.17, *p* < .001 for interpersonal stress; *b* = 0.91, SE = 0.21, *p* < .001 for non-interpersonal stress).

In line with previous work in young, physically healthy samples (Misener et al., 2008), the individual genetic polymorphisms did not predict depressive symptoms at age 20 (all *p* values > .10). Inflammatory genotypes were then examined as predictors of the magnitude of chronic stress exposure. *IL1β* genotype was found to predict chronic non-interpersonal stress exposure (*b* = -.36, SE = .14, *p* = .01), such that exposure increased with the number of minor alleles. There were no significant associations of *IL6* or *TNF* with either interpersonal or non-interpersonal stress expo sure (*p* values between .54 and .89).

### 3.1 Moderation of the depression and stress exposure relationship by genotype

In all models, maternal depression, age 15 BDI-II score, and exposure to chronic interpersonal stress remained significant as predictors of age 20 depressive symptoms following the addition of the main and interactive effects of genotype and stress (all *p*'s < .01, see Table 2 for interpersonal stress results).

#### 3.1.1 Moderation by IL6 genotype

**Fig. 1:**
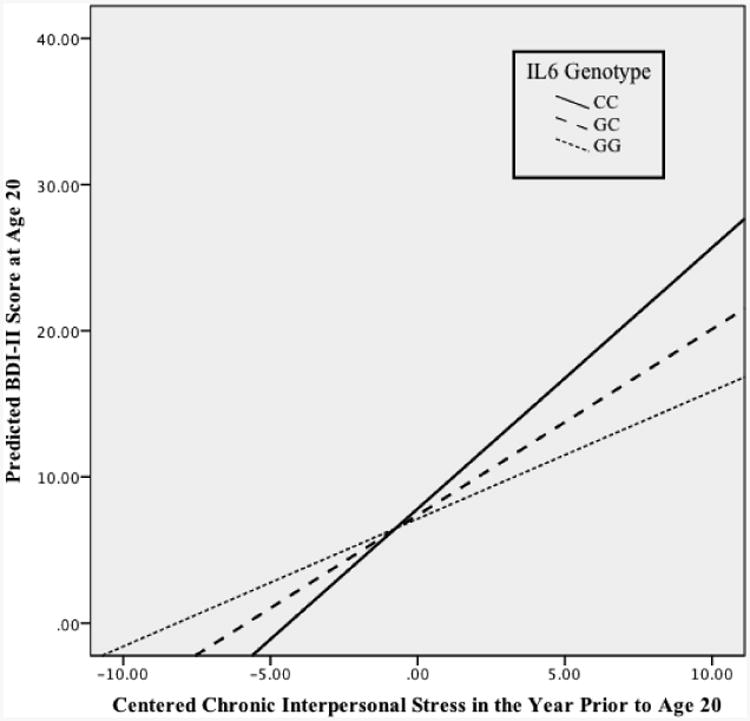
Allelic variation at -174G>C in *IL6* moderated the relationship between chronic interpersonal stress in the 6 months prior to age 20 and depressive symptoms at age 20 (*β* = -.14, *p* = .02). The slope of the regression line for CC homozygotes (solid line) was significantly different from the slope of the regression line for GG homozygotes (dotted line) (*b* = -0.98, SE = 0.42, *p* = 0.02). There was a marginally significant contrast in the slopes of the regression lines for CC homozygotes compared to heterozygotes (dashed line) (*b* = -0.58, SE = 0.31, *p* = 0.07). At the same levels of chronic interpersonal stress, CC homozygotes showed higher depressive symptoms compared to GG homozygotes.

Linear regression was used to determine whether allelic variation at *IL6* might moderate the impact of stress exposure on age 20 depression severity, over and above the effects of the covariates. An interaction of *IL6* genotype and chronic interpersonal stress exposure was detected and deconstructed into its simple effects (*b* = -.44 [95% CI: -.803 to -.078], Δ *R*^2^ = 0.01, Δ *F* = 5.71, *p* = .017, see Table 2). Post-hoc contrasts revealed significantly higher depressive symptoms in CC homozygotes compared with GG homozygotes (*b* = -0.98, SE = 0.42, *p* = 0.02), and marginally higher depressive symptoms in CC homozygotes compared with heterozygotes, given equal exposure to chronic interpersonal stress (*b* = -0.58, SE = 0.31, *p* = 0.07; see Figure 1). The contrast between GG 2and GC genotypes was not significant (*b* = 0.40, SE = 0.41, *p* = 0.34).

*IL6* genotype did not moderate the effects of chronic non-interpersonal stress on depressive symptoms at age 20 (*b* = -.36 [95% CI: -0.82 to 0.10], Δ *R*^2^ = 0.004, Δ *F* = 2.40, *p* = .12).

#### 3.1.2 Moderation by IL1β and TNF genotypes

**Fig. 2:**
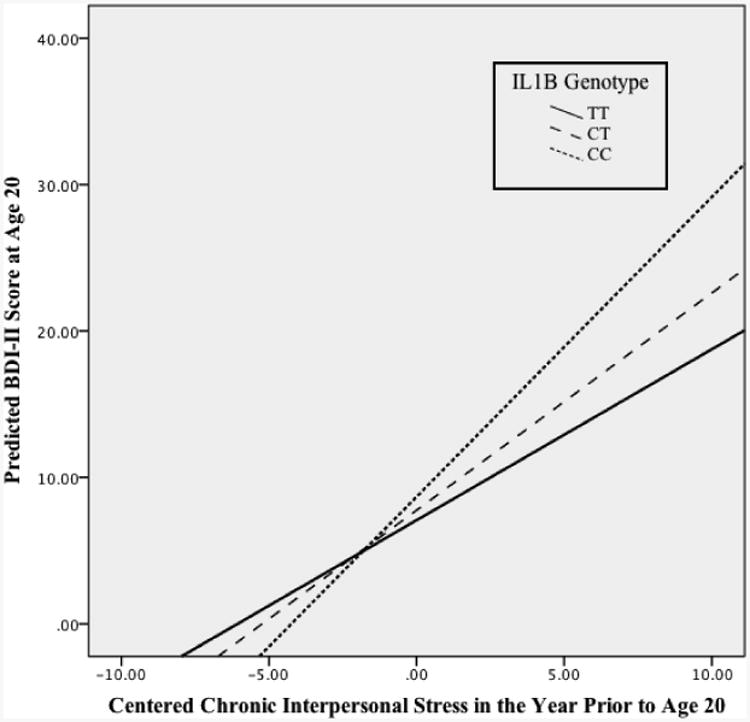
Allelic variation at -511 C>T in *IL1β* moderated the relationship between chronic interpersonal stress in the 6 months prior to age 20 and depressive symptoms at age 20 (*β* = 0.13, *p* = .022). The slope of the regression line for TT homozygotes (solid line) was marginally different from the slope of the regression line for CC homozygotes (dotted line) (*b* = 0.88, SE = 0.45, *p* = .053). There was also a marginally significant contrast in the slopes of the regression lines for TT homozygotes and heterozygotes (dashed line) (*b* = 0.59, SE = 0.31, *p* = .060).

The interaction of *IL1β* genotype and chronic interpersonal stress exposure predicted later depressive symptoms, even accounting for the effects of the covariates (*b* = 0.44, [95% CI: 0.06 to 0.82], Δ R^2^ = .009, Δ *F* = 5.29, *p* = .022, see Table 2 and Figure 2). Contrasts between genotypes at *IL1β* revealed marginally significant simple effects, such that CC homozygotes developed more depressive symptoms relative to TT homozygotes (*b* = 0.88, SE = 0.45, *p* = .05), and relative to heterozygotes (*b* = 0.59, SE = 0.31, *p* = .06).

Allelic variation at *IL1β* was not a significant moderator of the effects of non-interpersonal stress exposure on depressive symptoms (*b* = .43, SE = .26, *p* = .10).

Variation at *TNF* was not associated with differences in depressive symptoms as a function of chronic interpersonal stress (*b* = -.41, SE = 0.26, *p* = .11, see Table 2), but had a marginally significant effect on depressive symptoms in response to non-interpersonal stress (*b* = -0.60 [95% CI: -1.27 to 0.06], Δ *R^2^* = 0.005, Δ *F* = 3.18, *p* = .08). The simple effects of the *TNF* × non-interpersonal stress interaction were not significant (AA vs. GA: *b* = -0.69, SE = 2.01, *p* = 0.73; AA vs GG: *b* = -1.12, SE = 2.00, *p* = 0.57)^1^.

Following the primary analyses, we reran the models restricting the sample to Caucasians, the predominant ethnicity. The interaction effect of chronic interpersonal stress and *IL6* genotype on depression severity held under these conditions: *b* = -.48 [95% CI: -0.86 to -0.11], Δ *R*^2^ = 0.01, Δ *F* = 6.36, *p* = .01. Consistent with the results in the full sample, *IL6* genotype was not a moderator of the relationship between chronic non-interpersonal stress and depression (*b* = -.39, [95% CI: -0.87 to -0.08], Δ*R*^2^ = 0.01, Δ *F* = 2.63, *p* = .11). The interaction of chronic interpersonal stress and *IL1β* continued to predict depressive symptomatology: *b* = 0.49, [95% CI: 0.10 to 0.88], Δ R^2^ = .01, Δ *F* = 6.01, *p* = .02. When restricted to Caucasians, *IL1β* remained non-significant as a moderator of non-interpersonal stress, although the *p*-value was reduced to 0.08: *b* = 0.49, [95% CI: -0.05 to 1.03], Δ R^2^ = .01, Δ *F* = 3.17, *p* = .08. The interaction effects of *TNF* and chronic stress remained non-significant (*TNF* × interpersonal stress: *b* = -.45, SE = 0.27, *p* = .10, *TNF* × non-interpersonal stress: *b* = -0.58, SE = 0.35, *p* = .10). Analyses were repeated using similar stress and depression variables at the age 15 measurement time point, when the range in BDI-II symptoms (0-32) reflected lower severity of depression, compared with 0-52 at age 20, and no pattern of gene-environment interaction was identified [details available on request].

## 4. Discussion

The aim of the present study was to evaluate the interaction of chronic stress with three pro-inflammatory polymorphisms as precipitants of depressive symptoms in young adulthood. We hypothesized that variation at *IL6, IL1β* and *TNF* would potentiate the effects of interpersonal stress on the development of depressive symptoms. As hypothesized, interactive effects were revealed at loci in *IL6* and *IL1β* that accounted for differences in depressive symptoms, over and above the contributions of maternal depression history, earlier depressive symptoms, gender, and asthma diagnosis. These interactive effects were specific to interpersonal stress, as findings for non-interpersonal stress exposure were not significant. Findings were also specific to the age 20 time point. Of note, the range of BDI-II scores at age 15 (0-32) indicated lower severity of depression compared to age 20, when scores ranged from 0-52. A possibility is that the identified genetic effects emerge in late adolescence, which is in line with the antagonistic pleiotropy hypothesis generated by Cole et al. (2011) for the effects of *IL6*. Previous longitudinal work with twins has also found changes in genetic and environmental influences on depression (Lau & Eley, 2006; Kendler, Gardner, & Lichtenstein, 2008).

There were no main effects of genotype on depressive symptoms. Interestingly, the present study also found evidence of a gene-environment correlation at *IL1β*, so that exposure to non-interpersonal stress increased with each -511C allele. Further explication of this effect was not an aim of the present study and awaits future replication.

The pattern of results for *IL1β* is consistent with previous work that identified the high expression -511C allele as a risk for poor response to pharmacotherapies for depression, and for early onset of depression and higher depressive symptom severity (Dixon et al., 2007; Hwang et al., 2009; Tadic et al., 2008; Yu et al., 2003a). No previous empirical work has examined a gene-environment interaction at this locus, but the finding is in line with the pathogen host defense theory (PATHOS-D), which posits that pro-inflammatory genotypes enhance depression risk (Raison and Miller, 2012).

Results of analyses at *IL6* revealed that participants homozygous for -174C developed higher levels of depressive symptoms following exposure to chronic interpersonal stress when compared to those with one or more -174G alleles. This finding is consistent with a previous investigation that found elevations in plasma CRP following stress exposure in adolescents homozygous for -174C compared with -174G carriers (Cole et al., 2011). In contrast, -174G is associated with increased IL-6 expression following stress exposure in older adults (Cole et al., 2010; Schultz-Florey et al., 2012). To explain these conflicting results, Cole et al. (2011) hypothesize that -174G has undergone selection for its protection against stress-induced inflammation in adolescence, and becomes sensitized to stress after child-bearing age is reached. This is consistent with age-dependent antagonistic pleiotropy, as any detrimental effects of -174G on survival after reproductive age would not affect its retention in the population (Williams, 1957). No study has directly evaluated the hypothesis that aging leads to a change in the allele of risk at *IL6*, despite opposing findings in older adults and adolescents. Replication of the present results is needed, and further study should include measures of stress exposure, inflammatory markers and depressive outcomes in a single investigation.

Taken together, the present results suggest that possession of high expression alleles at *IL6* and *IL1β* increase depression risk following interpersonal stress exposure in the present sample. Further, these effects were specific to interpersonal stress and did not generalize across other types of adverse experience, potentially suggesting a specific biological vulnerability to interpersonal stress. Nesse (2009) has hypothesized that in our ancestry, interpersonal stress may have acted as a warning signal for physical confrontation or social abandonment. Individuals who were able to mount an early immune response to distressing social situations would have had an advantage in healing infections and wounds resulting from physical conflict or attack after the loss of community protection. In support of this theory, the biological changes induced by interpersonal stress mitigate injury and infection (Cole et al., 2007, 2011; Dhabhar, 2009). Although brief activation of the inflammatory response promotes healing, chronic activation leads to dysregulation of immunological processes and elicits depressive symptoms (Dhabhar, 2014; Dowlati et al., 2010; Howren et al., 2009; Raison and Miller, 2011). Theoretically then, individuals genetically predisposed to inflammation would be at increased risk for developing depressive illness when exposed to chronic interpersonal stress.

The present study was not designed to assess possible mechanisms linking stress exposure and inflammatory genotypes with depression. It may be that risk genotypes increase the production of pro-inflammatory cytokines in response to stress, which increases the risk of depression onset via action on neurotransmitter systems involved in depression (Dantzer et al., 2011; Myint et al., 2007). Consistent with this theory, findings from longitudinal studies in older populations indicate that rises in inflammation precipitate later depression (Matthews et al., 2007; Matthews et al., 2010; Pasco et al., 2010; Van Den Biggelaar et al., 2007). In never depressed youth, however, inflammatory levels were normative before depression onset, and a first depressive episode was found to increase inflammatory factors. Copeland et al. (2012) found that increases in pro-inflammatory cytokines arise as a consequence of depression onset in youth. Miller and Cole (2012) found that in young women, the first onset of a major depressive episode was accompanied by increases in plasma CRP and IL-6 that were more pronounced in women with higher levels of childhood stress. In women with a history of early stress, heightened inflammation persisted beyond the resolution of the MDE and predicted a second depressive episode. The role of age in determining the complex relationship between inflammation and depression is an important question for future research. In addition, future studies should explore the role of psychological resiliency and risk factors, including cognitive appraisal of stressful events and coping skills that may affect inflammatory changes and depression risk following stress exposure.

The present study had a number of strengths, including hypotheses based in theory, longitudinal assessment of the relationship between stress and depression, and validated, independently-rated measurement of chronic interpersonal and non-interpersonal stress using the semi-structured UCLA Life Stress Interview, which reduced the sample size needed to detect a significant interactive effect, and increased the validity of the findings (Caspi et al., 2010). Several alternative explanations for the present depression outcomes were ruled out, including effects of maternal depression, a factor that influences genetic makeup and psychosocial stress exposure, as well as prior elevations in depressive symptoms, gender and inflammatory illness (asthma).

Limitations of the present study include the retrospective assessment of chronic stress, and narrow age range of participants, which prevents a direct evaluation of the hypothesis that the allele of risk at *IL6* differs by age. The sample size was relatively small for a candidate gene study, which reduced our statistical power. The present study is an analysis of gene-environment interaction with specific a priori hypotheses dictated by theory, and we present the findings without correction for multiple analyses. Because of the limited sample size and restricted power, we present the analyses as an extension of previous work that identifies these SNPs as depression or stress-relevant. We readily acknowledge that the present results are not a conclusive description of the effects of the identified SNPs on the stress-depression relationship, as epistasis and other effects could also be relevant. Therefore, replication in a larger sample is necessary before any firm conclusions can be drawn from the present analyses.

When analyses were repeated within the largest ethnic group (Caucasians), the primary results held. Of interest, the interaction of *IL1β* with non-interpersonal stress remained non-significant, although a slightly larger difference between depression symptom means of allele groups (*p* reduced from .10 to .08) suggested that sample size may play a role in the magnitude of effects for different ethnicities. In fact, each of the gene × chronic stress effects moved closer to significance (*p*-values between .01-. 11) following the restriction to Caucasians, indicating that moderation by each genotype examined in the present study may be worth investigating further in larger samples. The small number of non-Caucasian subjects prevented an ethnically stratified analysis. Even considering these limitations, the present data are novel in finding that inflammatory genotypes moderate the effect of chronic interpersonal stress on depressive symptoms.

## Figures and Tables

**Table 1:** Socio-Demographic and Genetic Characteristics of Study Participants

	Males (*n* = 170)	Females (*n* = 250)
BDI-II Age 15 (SD)	5.20 (6.76)	6.30 (7.43)
BDI-II Age 20 (SD)	4.84 (6.08)	7.86 (9.71)
MDE at age 20 (yes/no)	12/158	31/219
Maternal Depression History (yes/no)	76/94	111/139
*IL6* -174G>C		
CC	61	95
GC	85	110
GG	24	45
*TNF* -308G>A		
AA	2	8
GA	43	73
GG	125	169
*IL1β*-511C>T		
TT	76	113
TC	72	115
CC	22	22
Asthma (yes/no)	12/155	23/222

**Table 2:** Hierarchical linear regression analyses of inflammatory genotypes, chronic interpersonal stress severity in the past 6 months, and their interactions, predicting depressive symptoms at age 20.

Predictor	Age 20 BDI-II Score			

	*b*	*SE*	*β*	*F change for interaction term*
***IL6* Model**				

Maternal Depression	2.01	0.65	3.09**	
Gender	0.51	0.65	0.78	
Asthma	-0.14	0.88	-0.01	
Age 15 BDI-II Score	0.41	0.05	0.32***	
Chronic Interpersonal Stress Age 20	1.39	0.19	0.43***	
*IL6*✠	-0.17	0.45	-0.02	
Chronic Interpersonal Stress Age 20*IL6	-0.44	0.18	-0.14*	5.71*

***IL1β* Model**				

Maternal Depression Gender	1.96 0.53	0.65 0.65	0.12** 0.03	
Asthma	-0.00	0.89	0.00	
Age 15 BDI-II Score	0.41	0.05	0.32***	
Chronic Interpersonal Stress Age 20	0.76	0.19	0.24***	
*IL1β*✠	0.38	0.49c	0.03	
Chronic Interpersonal Stress Age 20*IL1β	0.44	0.22	0.13*	5.29*

***TNF* Model**				

Maternal Depression	2.07	0.65	0.13**	
Gender	0.4*6*	0.65	0.03	
Asthma	-0.21	0.88	-0.01	
Age 15 BDI-II Score	0.41	0.05	0.32***	
Chronic Interpersonal Stress Age 20	1.74	0.46	0.54***	
*TNF*✠	0.56	0.60	0.04	
Chronic Interpersonal Stress Age 20*TNF	-0.41	0.26	-0.22	2.53

* *p* < .05,

** *p* < .01,

*** *p* < .001;

✠ Coded as 0/1/2 minor alleles

## Footnotes

1 Based on the results obtained with the individual genotypes, an exploratory analysis of additive risk at *IL6* and *IL1β* (-174C in *IL6* and -155T in *IL1β*) was conducted. A risk index was created with a range of 0-4 risk alleles, and tested as a moderator of chronic interpersonal stress at age 20, predicting BDI-II score, controlling for maternal depression, asthma, gender and age 15 BDI-II. The interaction of the genetic index and stress was significant (B = 0.45, SE = 0.14, *p* = .001), with each additional risk allele predicting greater mean depressive symptoms, *except* for participants who had zero risk alleles. Participants with zero risk alleles had the second lowest mean depression scores, between participants with one and participants with two risk alleles. When repeated in the sample's largest ethnic group (Caucasians), the interaction term held (B = .51, SE = .14, *p* < .001). Although this analysis had increased power to detect effects, it was not based on our original hypotheses, and for this reason, we do not include it as a primary analysis.
